# Effects of Natural Reduced Water on Cognitive Function, Body Composition, and Psychological Function in Older Adults: Study Protocol for a Randomized Controlled Trial

**DOI:** 10.3390/mps4040073

**Published:** 2021-10-14

**Authors:** Takamitsu Shinada, Yuji Takano, Keisuke Kokubun, Hikari Iki, Yasuyuki Taki

**Affiliations:** 1 Smart-Aging Research Center, Tohoku University, 4-1 Seiryo-Machi, Aoba-ku, Sendai 980-8575, Japan; yuji.takano.a6@tohoku.ac.jp (Y.T.); keisuke.kokubun.e3@tohoku.ac.jp (K.K.); yasuyuki.taki.c7@tohoku.ac.jp (Y.T.); 2 Department of Psychology, University of Human Environments, Matsuyama, Ehime 790-0825, Japan; 3 Department of Aging Research and Geriatric Medicine, Institute of Development, Aging and Cancer, Tohoku University, 4-1 Seiryo-Machi, Aoba-ku, Sendai 980-8575, Japan; iki.hikari.q6@dc.tohoku.ac.jp

**Keywords:** natural reduced water, diabetes, cognitive function, body composition, psychological function

## Abstract

Natural reduced water is natural water that contains active hydrogen and reduces oxidation. It is rare in the world, and in Japan, it is produced in the Hita area of Oita Prefecture (Hita Tenryosui water). Previous studies in humans have examined the effects of natural reduced water on diabetes, which is one of the known risks for dementia. Animal studies of natural reduced water have revealed anti-obesity and anti-anxiety effects. However, the effects of natural reduced water on cognitive function, body composition, and psychological function in humans are unknown. Therefore, we investigated the relationship between these items in elderly people who continuously consume natural reduced water. In this study, we recruited participants aged between 65 and 74 years. The participants were randomly and blindly assigned to a natural reduced water (Hita Tenryosui water) group or a control (tap water) group and drank 1 L of water daily for 6 months. Cognitive function, body composition, and psychological function were measured before and after the 6-month intervention period.

## 1. Introduction

**Figure 1:**
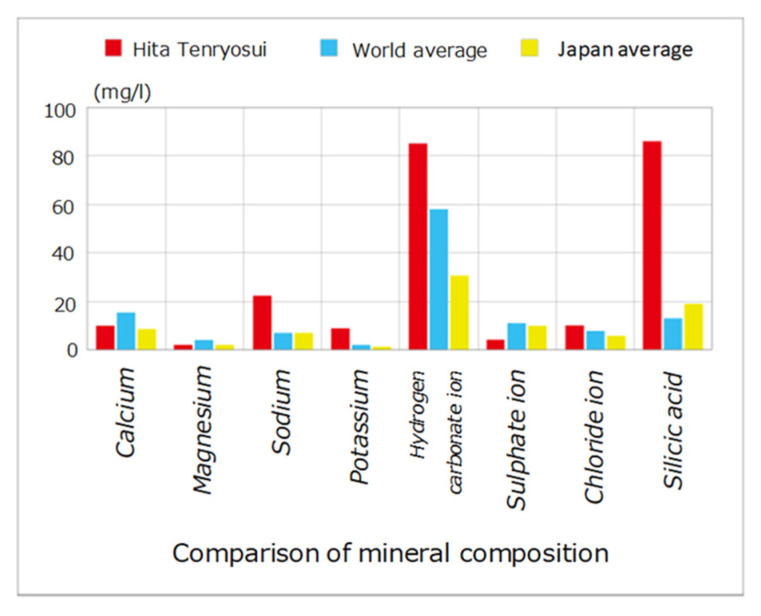
Comparison of mineral composition.

In the world, there are natural reduced waters that contain active hydrogen, such as Nordenau water in Germany and Tracote water in Mexico. In Japan, Hita Tenryosui water is known as natural reduced water, and is produced in the Hita Basin in northern Kyushu. Hita Tenryosui water is slightly alkaline (pH 8.3) and contains high concentrations of silicic acid, sodium, potassium, and hydrogen carbonate ion. The mineral composition of Hita Tenryosui water is shown in Figure 1.

Various risks of dementia have been considered, one of which is diabetes. In a meta-analysis of cohort studies, participants with diabetes had a significantly higher incidence of Alzheimer’s disease than those without diabetes [1]. In other meta-analyses, diabetes was associated with increased risks of progression of mild cognitive impairment (MCI) to dementia, and in participants with diabetes, a longer duration of diabetes and the presence of retinopathy were associated with an increased risk of progression from MCI to dementia [2]. In addition, the development of diabetes is associated with oxidative stress, and oxidative stress is a result of overproduction of oxidative-free radicals and associated reactive oxygen species (ROS) [3]. On the other hand, natural reduced water has been experimentally shown to scavenge ROS in cells [4]. The mechanisms of action of reduced water for scavenging ROS are considered to be complicated. Natural reduced water may have some active agents such as active hydrogen, hydrogen anions, hydrogen molecules, mineral nanoparticles, and mineral nanoparticle hydrides [5]. In an animal study, mice treated with alloxan, which promotes the development of diabetes, were administered natural reduced water, which suppressed their blood glucose levels [6]. Furthermore, in a human study, diabetic patients who consumed natural reduced water for 2 months had their fasting blood glucose levels reduced [6]. Therefore, we investigated the relationship between the maintenance or improvement of cognitive function and the consumption of natural reduced water in this study.

Previous research has shown that natural reduced water can improve symptoms in patients with hyperlipidemia [6]. In addition, in animal experiments, continuous drinking of natural reduced water showed anti-obesity effects [7]. There is evidence in clinical surveys linking obesity (or metabolic diseases) to gut microbiota fluctuation. Moreover, Hita Tenryosui water is known to change gut microbiota in animal experiments [7]. On the other hand, natural reduced water increased the expression of aquaporin, a membrane protein that specifically allows water molecules to pass through [8]. Aquaporins in the rat kidney have also been shown to play an important role in body water homeostasis [9]. Therefore, we hypothesized that natural reduced water would change body composition, such as body fat and body water content.

In addition, it has been confirmed that natural reduced water suppresses mental anxiety in mice [10]. When mice were fed natural reduced water for 6 months, urinary 8-hydroxy-2′-deoxyguanosine and blood urea nitrogen levels, which are used as biomarkers to detect anxiety, decreased. Moreover, natural reduced water exhibited anxiolytic-like effects in the conditioned fear and elevated plus maze tests. Hita Tenryosui water (natural reduced water) contains a lot of active hydrogen and reduced free radical production [4]. Stress related to anxiety was reported to be associated with increased free radical production, which natural reduced water may suppress [11]. In a 4-week human intervention experiment, natural killer (NK) cell activity was significantly higher in the group receiving natural reduced water than in the group receiving tap water [8]. Other previous studies have examined the relationship between NK cell activity and depressive tendencies. NK cell activity was reduced in depressed patients with a hip fracture compared with controls at 6 weeks post-fracture. Serum cortisol levels were also elevated in depressed patients compared with those in non-depressed patients [12]. Therefore, we examined the relationship between natural reduced water and psychological functions such as anxiety or stress level.

Based on these previous studies, we hypothesized that the continuous consumption of natural reduced water may affect cognitive function, body composition, and psychological function, and we tested this hypothesis.

## 2. Methods

### 2.1. Study Design

This study was a double-blind, randomized controlled trial that employed a design to measure cognitive function before and after a 6-month intervention for the natural reduced water and tap water groups. The purpose of this study was to determine the relationship between the continuous consumption of natural reduced water and cognitive function, body composition, and psychological function. In terms of cognitive function, we assessed executive function, attention function, short-term memory, and verbal fluency. In addition, we tested body composition (e.g., body fat and body water content) and psychological function (e.g., anxiety and stress levels).

### 2.2. Patient and Public Involvement

We recruited participants for this experiment by advertising in a local newspaper. This local newspaper is a weekly publication with a circulation of 450,000 in the Sendai area (Miyagi Prefecture). As this study is aimed at preventing dementia, we focused our recruitment on the age group where most elderly people without dementia are concentrated.

### 2.3. Inclusion Criteria

The inclusion criteria were as follows: (1) age between 65 and 74 years old and (2) healthy elderly people who live independently in daily life.

### 2.4. Exclusion Criteria

Based on previous studies [13,14,15,16], the exclusion criteria for the participants included conditions related to electrolyte abnormalities (such as hyponatremia). The criteria were as follows:

(1) People with a current medical history:
  – Renal disease;
  – Cardiac disease;
  – Liver cirrhosis;
  – Syndromes of inappropriate antidiuretic hormone (SIADH);
  – Hypothyroidism;
  – Diabetes;
  – Hyperlipidemia;
  – Mental illness (polydipsia);
  – Vomiting or diarrhea.
(2) People taking oral medications:
  – Antiepileptic drugs;
  – Antidepressants;
  – Diuretics;
  – Antihypertensive agents;
  – Antiarrhythmic drugs.
(3) People with severe visual or hearing impairment;
(4) People who have continuously consumed natural reduced water in the last year.

### 2.5. Water Used for the Intervention

The natural reduced water used in this study was commercially available Hita Tenryosui water. For tap water, we used tap water from Oita Prefecture that had been boiled. Each type of water was poured into plastic bottles at the factory of Hita Tenryo-Sui Co., Ltd. The plastic bottles for both types of water were not labeled, and the same caps were used so that the participants themselves would not know which type of water they were drinking. Each bottle of water was delivered to the participant’s home on a regular basis.

### 2.6. Protocol

**Figure 2:**
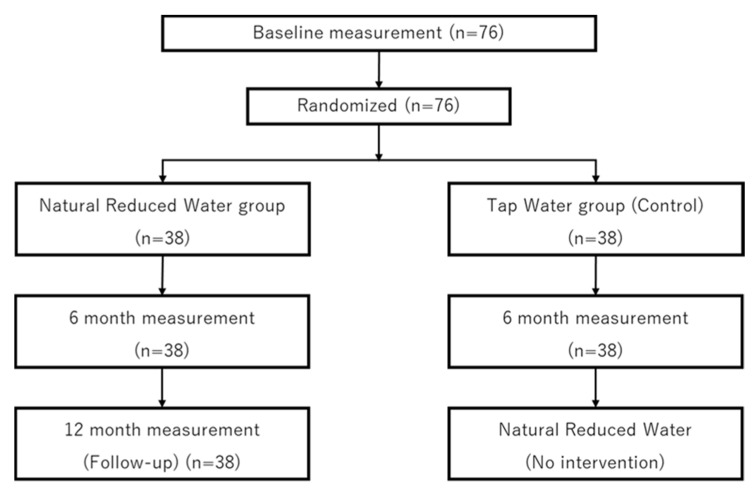
Flowchart of the natural reduced water intervention study.

The design of this study is illustrated in Figure 2. First, we explained the contents of this study to the participants and obtained their informed consent. We then administered a pre-intervention baseline test to the participants. In this test, we measured cognitive function, body composition, and psychological function. The participants were then randomly divided into the natural reduced water group and tap water group, and the intervention was conducted for 6 months. During the intervention period, participants consumed 1 L of each group’s water daily and wrote down their intake on a list. We would have preferred to set the daily consumption of water to 2 L as in previous studies of Hita Tenryosui water [4], but considering the risk of hyponatremia in internal discussions, we decided to set the amount as 1 L per day. The participants were blinded so that they could not tell which water they were drinking. After the 6-month intervention, a post-intervention examination was conducted. We administered a pre-test and post-test before and after the 6-month period, drawing on other intervention studies of cognitive function [17,18]. This study will investigate the maintenance or improvement of cognitive function in healthy elderly people, not in people with dementia or other illnesses. In addition, previous studies have shown significant improvement in cognitive function in a 6-month intervention study [17,18]. The content of the post-intervention test was the same as that of the pre-intervention test. After the post-intervention test, the participants in the natural reduced water group had a follow-up test 6 months later (12 months after the start of the intervention) and were told not to drink the natural reduced water until the follow-up test. The participants in the tap water group were asked if they wanted to drink natural reduced water, and those who wanted to could drink the same amount as the natural reduced water group after the post-intervention test. Data from people whose water intake is less than 90% of the total will be excluded as dropped data. We will also exclude people who become unable to drink water during the course of the intervention or who start taking medications that meet the exclusion criteria.

### 2.7. Measurement Items

The measurement items were as follows:

(1) Mini-Mental State Examination (MMSE) [19]: The MMSE is a 30-point paper-based test that is used to measure global cognitive function (cut-off point, 23/24).
(2) Trail Making Test (TMT) [20]: The TMT is used to measure executive function. This test is a commonly used method for testing attention function. The test consisted of two parts. In part A, participants were instructed to connect 26 numbered circles in ascending order. In part B, participants were instructed to connect numbers and letters in an alternating fashion. The time taken was then measured.
(3) Digit Span (DS) [21]: The DS is a subtest from the Wechsler Adult Intelligence Scale-III to assess short-term memory. In this test, the participants had to answer the spoken numbers in the same order. Subsequently, they had to answer in reverse order.
(4) Verbal Fluency Test (VFT) [22]: The VFT is used to assess verbal fluency. For the VFT, participants had to write as many words as possible in 60 s. The test consisted of two tasks. The first task was to write a word of a specified category, such as fruits. The second task was to write a word that started with a specified letter.
(5) Profile of Mood States 2 (POMS 2) [23]: The POMS 2 assesses the mood states of individuals 13 years of age and older. It yields several scale scores: anger–hostility, confusion–bewilderment, depression–ejection, fatigue–inertia, tension–anxiety, and vigor–activity. We used the shortened version, and there were 35 items.
(6) Perceived Stress Scale (PSS) [24]: The PSS is the most widely used psychological instrument for measuring the perception of stress. It is a measure of the degree to which situations in one’s life are appraised as stressful and consists of 14 items.
(7) Subjective Happiness Scale (SHS) [25]: The SHS is a four-item scale of global subjective happiness. Two items ask respondents to characterize themselves using both absolute ratings and ratings relative to peers, whereas the other two items offer brief descriptions of happy and unhappy individuals and ask respondents the extent to which each characterization describes them.
(8) Body Composition: We measured the participants’ body composition using a body composition analyzer (RD-800; TANITA, Japan). The measurement items included body water content, body fat, and muscle mass.

### 2.8. Sample Size

Assuming a significance level (α) of 5%, the effect size at 0.6, and a power of test (1 − β) of 80%, which are all “moderate” levels according to Cohen, the required number of participants was 34 in each group. Six months of intervention, assuming a drop rate of 10%, would require approximately 38 participants, for a total of 76 recruits. We added two more people to each group in case of unforeseen circumstances.

### 2.9. Statistical Considerations

We conducted a two-factor analysis of variance with group (intervention and control groups) and time (pre-intervention and post-intervention) as factors to examine the relationship between the scores of each measurement item in the intervention and control groups. Using path analysis, we plan to conduct an exploratory analysis to investigate the relationship between each measurement item.

## 3. Expected Results

We expect that continuous consumption of natural reduced water will improve cognitive function in the elderly. We also predict that body water content will increase and body fat percentage will decrease in the body composition of the participants. Furthermore, it is expected that anxiety and stress levels will decrease and subjective happiness will increase.

## 4. Discussion

Although various effects of natural reduced water (Hita Tenryosui water) have been studied in animal experiments, the relationship between natural reduced water and cognitive function in humans has not yet been investigated, so this research may be a novel study. There have been various studies on the prevention of dementia; this study examines the use of water, which is essential in daily life, as a potentially easy way to prevent dementia. On the other hand, one of the limitations of this study is that participants were free to consume water other than the daily 1 L of water in the study; thus, there could be effects from these other types of water, but this was true for both the intervention and control groups. Since this study was conducted on Japanese people, the applicability of this study to people of other nationalities needs to be verified in another study.

## Data Availability

The data obtained in this study will be anonymized and managed with a research ID. The results of the study will be published in conferences and scientific journals, but no personal information will be disclosed. There is a possibility that the research data will be used for secondary purposes in other research in the future, but in that case, it will be used only after approval by the Institutional Review Board.

## References

[1] Zhang J., Chen C., Hua S., Liao H., Wang M., Xiong Y., Cao F. (2017). An updated meta-analysis of cohort studies: Diabetes and risk of Alzheimer’s disease. Diabetes Res. Clin. Pract..

[2] Pal K., Mukadam N., Petersen I., Cooper C. (2018). Mild cognitive impairment and progression to dementia in people with diabetes, prediabetes and metabolic syndrome: A systematic review and meta-analysis. Soc. Psychiatry Psychiatr. Epidemiol..

[3] Newsholme P., Cruzat V.F., Keane K.N., Carlessi R., de Bittencourt P.I.H. (2016). Molecular mechanisms of ROS production and oxidative stress in diabetes. Biochem. J..

[4] Li Y., Nishimura T., Teruya K., Maki T., Komatsu T., Hamasaki T., Shirahata S. (2002). Protective mechanism of reduced water against alloxan-induced pancreatic β-cell damage: Scavenging effect against reactive oxygen species. Cytotechnology.

[5] Shirahata S., Hamasaki T., Teruya K. (2012). Advanced research on the health benefit of reduced water. Trends Food Sci. Technol..

[6] Osada K., Li Y., Hamasaki T., Abe M., Nakamichi N., Teruya K., Shirahata S. (2010). Anti-diabetes effects of Hita Tenryou-Sui Water^®^, a natural reduced water. Animal Cell Technology. Basic & Applied Aspects.

[7] Yahiro T., Hara T., Matsumoto T., Ikebe E., Fife-Koshinomi N., Xu Z., Inomata M. (2019). Long-term potable effects of alkalescent mineral water on intestinal microbiota shift and physical conditioning. Evid.-Based Complementary Altern. Med. eCAM.

[8] Kitagawa Y., Liu C., Ding X. (2011). The influence of natural mineral water on aquaporin water permeability and human natural killer cell activity. Biochem. Biophys. Res. Commun..

[9] Kwon T.H., Frøkiær J., Nielsen S. (2013). Regulation of aquaporin-2 in the kidney: A molecular mechanism of body-water homeostasis. Kidney Res. Clin. Pract..

[10] Masuda K., Tanaka Y., Kanehisa M., Ninomiya T., Inoue A., Higuma H., Akiyoshi J. (2017). Natural reduced water suppressed anxiety and protected the heightened oxidative stress in rats. Neuropsychiatr. Dis. Treat..

[11] Hassan W., de Castro Gomes V., Pinton S., da Rocha J.B.T., Landeira-Fernandez J. (2013). Association between oxidative stress and contextual fear conditioning in Carioca high-and low-conditioned freezing rats. Brain Res..

[12] Duggal N.A., Upton J., Phillips A.C., Hampson P., Lord J.M. (2015). NK cell immunesenescence is increased by psychological but not physical stress in older adults associated with raised cortisol and reduced perforin expression. Age.

[13] Verbalis J.G., Goldsmith S.R., Greenberg A., Schrier R.W., Sterns R.H. (2007). Hyponatremia treatment guidelines 2007: Expert panel recommendations. Am. J. Med..

[14] Filippatos T.D., Makri A., Elisaf M.S., Liamis G. (2017). Hyponatremia in the elderly: Challenges and solutions. Clin. Interv. Aging.

[15] Intravooth T., Staack A.M., Juerges K., Stockinger J., Steinhoff B.J. (2018). Antiepileptic drugs-induced hyponatremia: Review and analysis of 560 hospitalized patients. Epilepsy Res..

[16] Grattagliano I., Mastronuzzi T., D’Ambrosio G. (2018). Hyponatremia associated with long-term medication use in the elderly: An analysis in general practice. J. Prim. Health Care.

[17] Uenobe M., Saika T., Waku N., Ohno M., Inagawa H. (2019). Efficacy of continuous ingestion of dewaxed brown rice on the cognitive functions of the residents of elderly welfare facilities: A pilot test using crossover trial. Food Sci. Nutr..

[18] Hariprasad V.R., Koparde V., Sivakumar P.T., Varambally S., Thirthalli J., Varghese M., Basavaraddi I.V., Gangadhar B.N. (2013). Randomized clinical trial of yoga-based intervention in residents from elderly homes: Effects on cognitive function. Indian J. Psychiatry.

[19] Folstein M.F., Folstein S.E., McHugh P.R. (1975). ‘Mini-mental state’: A practical method for grading the cognitive state of patients for the clinician. J. Psychiatr. Res..

[20] Reitan R.M. (1992). Trail Making Test: Manual for Administration and Scoring.

[21] Fujita K., Maekawa H., Dairoku H., Yamanaka K. (2006). Japanese Wechsler Adult Intelligence Scale.

[22] Lezak M.D., Howieson D.B., Loring D.W., Hannay H.J., Fischer J.S. (2004). Neuropsychological Assessment.

[23] Konuma H., Hirose H., Yokoyama K. (2015). Relationship of the Japanese translation of the profile of mood states second edition (POMS 2^®^) to the first edition (POMS^®^). Juntendo Med. J..

[24] Sumi K. (2006). Reliability and validity of the Japanese version of the Perceived Stress Scale. Jpn. J. Health Psychol..

[25] Shimai S., Otake K., Utsuki N., Ikemi A., Lyubomirsky S. (2004). Development of a Japanese version of the Subjective Happiness Scale (SHS), and examination of its validity and reliability. Jpn. J. Public Health.

